# An Australian survey of health professionals’ perceptions of use and usefulness of electronic medical records in hospitalised children’s pain care

**DOI:** 10.1177/13674935241256254

**Published:** 2024-05-29

**Authors:** Nicole Pope, Janelle Keyser, Dianne Crellin, Greta Palmer, Mike South, Denise Harrison

**Affiliations:** 1 6453 Department of Nursing Research, The Royal Children’s Hospital, Melbourne, VIC, Australia; 2 6453 Department of Nursing, Melbourne School of Health Sciences Faculty of Medicine, Dentistry, and Health Sciences, The University of Melbourne, Melbourne, VIC, Australia; 3 The Murdoch Children’s Research Institute, Melbourne, VIC, Australia; 4 Child Health Evaluative Services, Research Institute, The Hospital for Sick Children, Toronto, ON, Canada; 5 6453 Acute Pain Service, Queensland Children’s Hospital and Health Service, Brisbane, QLD, Australia; 6 Department of Anaesthesia and Pain Management, The Royal Children’s Hospital, Melbourne, VIC, Australia; 7 Emergency Department, The Royal Children's Hospital, Melbourne, VIC, Australia; 8 Department of Anaesthesia and Pain Management, The Royal Children's Hospital, Melbourne, VIC, Australia; 9 Department of Paediatrics, Melbourne School of Health Sciences Faculty of Medicine, Dentistry, and Health Sciences, The University of Melbourne, VIC, Australia; 10 Department of General Medicine, The Royal Children's Hospital, Melbourne, VIC, Australia; 11 Faculty of Health Sciences, The University of Ottawa, Ottawa, ON, Canada

**Keywords:** Pain, family-centred care, paediatric, electronic health records

## Abstract

Pain in hospitalised children is common, yet inadequately treated. Electronic medical records (EMRs) can improve care quality and outcomes during hospitalisation. Little is known about how clinicians use EMRs in caring for children with pain. This national cross-sectional survey examined the perceptions of clinician-EMR users about current and potential use of EMRs in children’s pain care. One hundred and ninety-four clinicians responded (*n* = 81, 74% nurses; *n* = 21, 19% doctors; *n* = 7, 6% other); most used Epic (*n* = 53/109, 49%) or Cerner (*n* = 42/109, 38%). Most (*n* = 84/113, 74%) agreed EMRs supported their initiation of pharmacological pain interventions. Fewer agreed EMRs supported initiation of physical (*n* = 49/113, 43%) or psychological interventions (*n* = 41/111, 37%). Forty-four percent reported their EMR had prompt reminders for pain care. Prompts were perceived as useful (*n* = 40/51, 78%). Most agreed EMRs supported pain care provision (*n* = 94/110, 85%) and documentation (*n* = 99/111, 89%). Only 39% (*n* = 40/102) agreed EMRs improved pain treatment, and 31% (*n* = 32/103) agreed EMRs improved how they involve children and families in pain care. Findings provide recommendations for EMR designs that support clinicians’ understanding of the multidimensionality of children’s pain and drive comprehensive assessments and treatments. This contribution will inform future translational research on harnessing technology to support child and family partnerships in care.

## Introduction

### Background

Most hospitalised children, especially the youngest and sickest, experience pain that is often severe yet inadequately treated (Cruz et al., 2016; Plummer et al., 2021; Senger et al., 2021). Although pain care has dramatically improved in the last 30 years (Eccleston et al., 2021), pain remains a persistent problem for hospitalised children (Friedrichsdorf and Goubert, 2020; Holm et al., 2023). A systematic review of 18 epidemiological studies from 13 countries reported that critically unwell neonates undergo up to 17 painful procedures per day, most performed without analgesia (Cruz et al., 2016). Children with severe or chronic medical conditions also suffer inadequately treated disease-related pain and pain from invasive procedures (Fortier et al., 2020; Plummer et al., 2021).

The undertreatment of children’s pain is disturbing, given that it is a strong predictor of adverse short and life-term outcomes that impact children, their families, and communities. Undertreated pain is linked to delayed recovery (Williams et al., 2015), prolonged hospitalisations, and increased complications (Rosenbloom et al., 2021). Repeated pain exposure in premature infants may impact neurodevelopment and future pain responses in childhood and adulthood (Valeri et al., 2016; Williams and Lascelles, 2020) and lead to needle phobias and medical care avoidance (McMurtry et al., 2015, 2016). Despite the long-standing guidance on effective pain management, there is little evidence of sustained practice change and improved outcomes (Eccleston et al., 2021).

The use of digital technology to support high-quality care, safe practices, and improved outcomes is an emerging area in healthcare (Antonio et al., 2020; Crowley et al., 2019). Digital solutions have already influenced children’s pain in ambulatory settings. For example, in Canada, the *iCanCope with Pain* platform supports youth to self-manage persistent pain conditions (Birnie et al., 2019). In Australia, the pain*HEALTH* website provides a digital resource supporting young people with musculoskeletal pain (Slater et al., 2020).

### The current study

In paediatric hospitals, growing evidence demonstrates that electronic medical records (EMRs) and patient portals (electronic personal health record applications linked to EMRs) can improve adherence to best-practice guidelines (Horton et al., 2020; McGreevey et al., 2020; Sutton et al., 2020), child and family engagement (Antonio et al., 2020; Bush et al., 2016), and outcomes (Antonio et al., 2020; South et al., 2022). Paediatric pain experts (Pope et al., 2023), children, and families (Kelly et al., 2019a, 2019b) also advocate for digital technologies co-designed with these stakeholders and tailored to their needs and contexts to improve pain care for hospitalised children. But not much is known about how health professionals use EMRs in the daily care of children with pain.

### Aim

This national survey aimed to measure health professional perceptions of use and usefulness of EMRs in Australian tertiary paediatric hospitals to enhance understanding of current and potential use of EMRs in children’s pain care.

## Methods

### Research design

A descriptive, cross-sectional online survey hosted on the Qualtrics^XM^ (Qualtrics, Provo, UT, USA) platform was used to collect quantitative information from clinicians. The survey was designed specifically for this study, with design input from Australian clinicians.

### Data collection tools

The 14-item survey comprised questions about pain assessments and treatments using EMRs, perceptions about how EMRs influenced pain care, and respondent demographic characteristics. Survey items were multiple choice and fixed questions formatted as 5- or 7-item Likert scales. Open-ended questions allowed respondents to elaborate on their responses. No survey items were mandatory. Respondents could modify entries before submission and exit the survey anytime.

Researchers from Australia developed the survey, and initial testing was conducted with five expert clinicians who had experience treating children with pain, building surveys, and/or working with EMRs. The survey was conducted from October to December 2022. Reporting follows the Checklist for Reporting Results of Internet E-Surveys (Eysenbach, 2004).

### Sample and recruitment

The anonymous online survey was distributed via email by representative heads of clinical departments at the five participating hospitals. The email included a weblink to the survey, comprising study information, electronic consent, and the questionnaire.

English-speaking registered nurses and medical doctors (aggregated as ‘clinicians’) working in various clinical areas at one of the five Australian tertiary paediatric hospitals with a comprehensive EMR were targeted. A comprehensive EMR was defined as a system comprising the essential functions to support pain care, such as electronic documentation of vital signs, pain assessment and documentation of pain care-related interventions, and order entry for actionable items. Five of the nine Australian tertiary paediatric hospitals were included as they have this level of EMR. They are in major Australian cities in the eastern states. One of the five hospitals had used its EMR for 6 years, and the remaining hospitals introduced their EMR less than 5 years before the study.

### Analysis

The response rate was calculated as the number of participants completing consent and commencing the survey divided by the estimated potential respondents (*n* = ∼750) who comprised the total sample group. The study is descriptive, and therefore, inferential analysis was not conducted. We used descriptive statistics (percentages and frequencies) and Statistical Package for Social Sciences (SPSS Inc., Chicago, IL, USA) for Windows (Version 22, 2013) for analysis. Due to very few responses in strongly and somewhat categories, 7-Likert scale responses were consolidated into three categories: (1) ‘agreed’ (combining strongly agree, agree, and somewhat agree) or ‘useful’ (combining extremely useful, moderately useful, and slightly useful) (2) ‘neutral’ (neither agree nor disagree or neither useful/useless) and (3) disagree (combining the remaining categories). All survey item responses were included and individually reported, including partially completed questionnaires.

### Ethical aspects

Survey protocols followed Helsinki guidelines. This research was approved by the University of Melbourne’s Human Research Ethics Committee (ID: 2022-23409-30913-3), establishing the procedures and regulatory rules for human research. Participants were not offered remuneration and clinicians agreed to participate in the study by completing and submitting the online survey. A brief written information sheet was provided with a link to the online survey, which contained contact details for the research team if participants had questions or required more information. The study information made it clear to participants that they were providing permission (consent) for their answers to be used as part of the study by submitting their answers.

## Results

### Respondent characteristics

A total of 194 health professionals working in one of the five hospitals with comprehensive EMRs consented to participate (response rate ∼26%). Not all respondents completed every question. Very few respondents (*n* = 6, 3.1%) provided open-ended text responses; these data are integrated as illustrative quotes in results reporting. Table 1 reports respondent characteristics. Most (*n* = 81/109, 74%) were nurses, and over half (*n* = 64/110, 58%) were from the two Melbourne centres. They were mostly experienced clinicians, with 69 out of 110 (63%) having more than 10 years of clinical experience and half (*n* = 57/110, 52%) having more than 10 years of experience in paediatric healthcare. Most respondents had experience using Epic (https://www.epic.com/software) *n* = 53/109 49% or Cerner (https://www.cerner.com/solutions/health-systems) *n* = 42/109, 38% systems (See Table 1).

**Table 1.** Respondent characteristics of clinicians working at five Australian tertiary paediatric centres with comprehensive EMRs.

Characteristic	Value *n* (%)
Profession	*n* = 109
Nursing	81 (74%)
Registered nurse	75 (69%)
Nurse practitioner	4 (4%)
Clinical nurse consultant/educator	2 (2%)
Medical doctor	21 (19%)
Paediatrician	13 (12%)
Anaesthesiologist	4 (4%)
Neonatologist	2 (2%)
Registrar (trainee in residency program)	2 (2%)
Other (did not specify)	7 (6%)
City location of the tertiary paediatric hospital	*n* = 110
Melbourne (Royal Children’s Hospital; Monash Children’s Hospital)	64 (58%)
Brisbane (Queensland Children’s Hospital)	32 (29%)
Sydney (Sydney Children’s Hospitals; Westmead and Randwick)	14 (13%)
Years of experience as a registered health professional	*n* = 110
More than 20 years	28 (26%)
11-20 years	41 (37%)
5-10 years	22 (20%)
Less than 5 years	19 (17%)
Years of experience in paediatric health care	*n* = 110
More than 20 years	24 (22 %)
11-20 years	33 (30%)
5-10 years	28 (26%)
Less than 5 years	25 (23%)
Reported use of EMR type	*n* = 109
Epic	53 (49%)
Cerner	42 (38%)
MetaVision	4 (4%)
Other	10 (9%)

### EMRs in pain assessment

**Figure 1.**
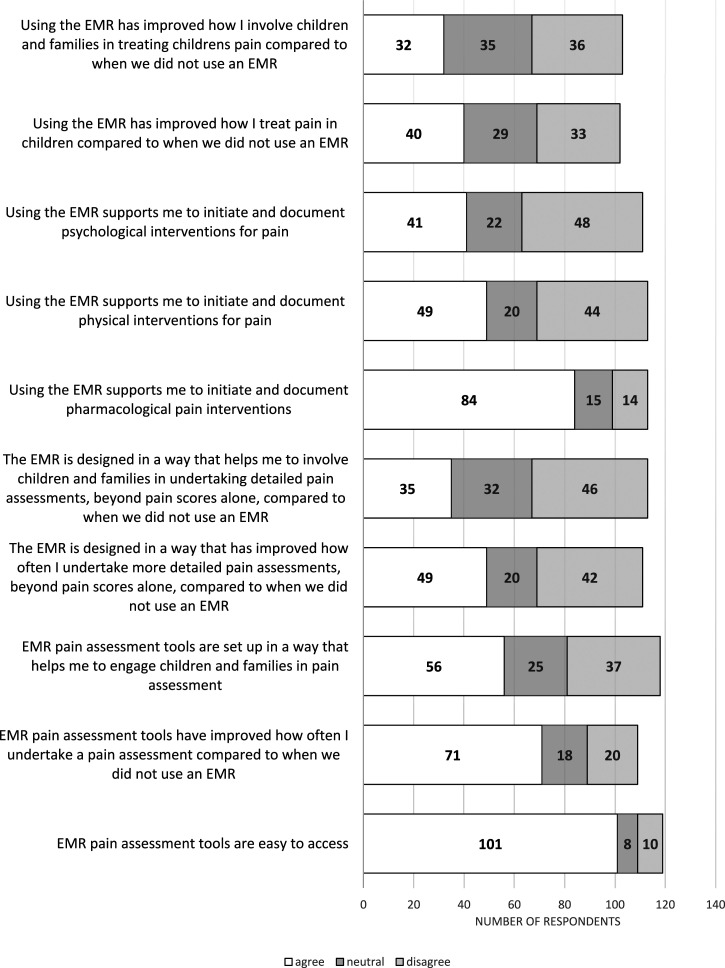
Distribution of ratings of perceived agreement with influence of EMR on components of pain care practice from clinicians using EMRs (*n* = 103–119).

Respondents reported 14 pain assessment tools integrated into their EMRs (Table 2). The most common were the Face, Legs, Activity, Cry, Consolability Scale, Numerical Rating Scale- Revised, and the Wong Faces Pain Scale. As seen in Figure 1, most respondents (i) (*n* = 101/119, 84%) agreed the EMR pain assessment tools were easy to access; (ii) agreed (*n* = 71/109, 65%) EMR pain assessment tools improved how often they undertook pain assessment compared to when they did not use an EMR; and (iii) used the pain assessment fields to record pain scores (*n* = 86/129, 66%), while one-quarter (*n* = 32/129, 25%) still recorded pain scores in free-text fields.

**Table 2.** Paediatric pain assessment scales used in comprehensive EMRs at five Australian tertiary paediatric centres.

Pain scale	Frequency *n* = 194 (%)
Face, Legs, Activity, Cry, Consolability Scale	90 (69.8)
Numerical Rating Scale	75 (58.1)
Wong-Baker Faces Pain Rating Scale	66 (51.2)
Modified Pain Assessment Tool	32 (24.8)
Neonatal Pain Assessment Tool	21 (16.3)
Faces Pain Scale- Revised	20 (15.5)
Functional Activity Score	20 (15.5)
Comfort B	18 (14)
Visual Analogue Scale	10 (7.8)
Premature Infant Pain Profile	8 (6.2)
Neonatal Pain, Agitation, Sedation Scale	8 (6.2)
Face, Legs, Activity, Cry, Consolability- Revised Scale	8 (6.2)
Comfort Analogue Scale	2 (1.6)
Premature Infant Pain Profile Revised	1 (0.8)

### EMRs in pain treatment

#### Pharmacological treatments

**Figure 2.**
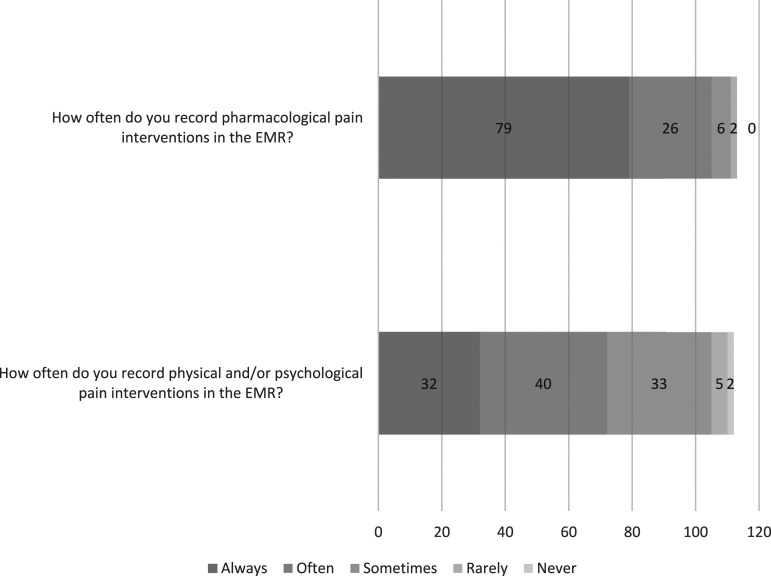
Distribution of ratings of frequency of pain treatment documentation from clinicians using EMRs (*n* = 112-113).

Most (*n* = 84/113, 74%) agreed the EMR supported them in initiating and documenting pharmacological pain interventions (i.e. medications, including sucrose) (Figure 1). Regarding documentation frequency, most (*n* = 79/113, 70%) reported always recording pharmacological interventions in the EMR (Figure 2). The majority (*n* = 96/113, 85%) recorded these in the electronic medication administration record (eMAR); most (*n* = 83/113, 73.5%) also recorded pharmacological interventions in their nursing or medical electronic progress notes (i.e. they duplicate recorded).

#### Physical and psychological treatments

Less than half (*n* = 49/113, 43%) agreed that the EMR supported them in initiating and documenting physical interventions for pain (i.e. comfort positioning, breastfeeding, skin-to-skin, and heat/ice packs) (Figure 1). Fewer (*n* = 41/111, 37%) agreed that the EMR supported them in initiating and documenting psychological interventions (i.e. distractions such as games, music, iPad, TV, and reading). Regardless, the majority (*n* = 89/113, 79%) of respondents stated they still usually recorded physical interventions, and many recorded psychological interventions (*n* = 76/113, 67%) and interdisciplinary referrals (i.e. occupational therapist, music therapist, life specialist, and pain team) (*n* = 73/113, 65%). The majority of respondents recorded non-medication interventions in progress notes (*n* = 91/113, 81%), almost a third (*n* = 32/113, 28%) reported using the pain assessment fields, and fewer (*n* = 20/113, 18%) recorded this in vital signs fields. However, only 29% (*n* = 32/112) reported they always recorded these interventions in the EMR (Figure 2).

#### Prompts

Less than half of respondents (*n* = 51/116, 44%) reported that their EMR had prompts or alerts to remind them to undertake a pain assessment or initiate medications. Only 8% (*n* = 9/112) reported prompts for other pain interventions (physical and psychological). Of the 51 respondents whose EMR had the capacity for prompts (*n* = 40/51, 78%), most agreed these were useful. However, some suggested that active prompts were unnecessary, *‘I just assess the pain regardless of if I’m prompted or not’*. Task lists and checklists were prompts in themselves that ‘*improved the visibility of required actions’.* Some also suggested the need to balance prompts to prevent *‘prompt/click fatigue’.*

#### Overall influence and suggestions for EMRs in pain care

**Figure 3.**
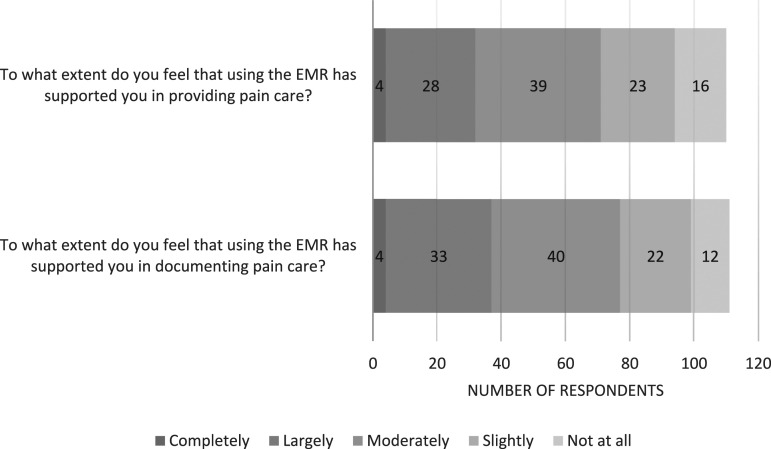
Distribution of perceived impact of EMR on pain care and documentation by clinicians using EMRs (*n* = 110-111).

The majority agreed that using the EMR supported them in pain care provision (*n* = 94/110, 85%) and documentation (*n* = 99/111, 89%) (Figure 3), yet fewer respondents (*n* = 40/102, 39%) agreed that the EMR improved how they treat children’s pain compared to when they did not use an EMR (Figure 1). Even fewer (*n* = 32/103, 31%) agreed that the EMR improved how they involve children and families in treating children’s pain compared to when they did not use an EMR. There were suggestions about the potential for patient-facing portable devices and *‘client/patient portal views’* to *‘empower’* and ‘*give ownership’* to patients and families.

## Discussion

The results provide pertinent perspectives on current practices and potential use of EMRs in children’s pain care reported by a diverse sample of experienced nurses and medical clinicians. Australia has been working on improving pain care in children for a long time, and implementation of EMRs in hospitals offers promise to improve pain care practices (Australian Digital Health Agency (ADHA), 2019). Given their years of experience, respondents were likely, before the EMR, to have used paper-based systems for children’s pain care and therefore had capacity to compare pain care practices before and after EMR implementation (ADHA, 2019). A key finding from this work is that clinicians perceived that EMRs had little overall influence on the pain care they provided to hospitalised children compared to when they used paper-based systems. This aligns with other studies describing clinician scepticism regarding the effect of EMRs on their provision of care in adult settings (Crowley et al., 2019; Emani et al., 2017). It might reflect an expectation and experience gap between the benefits of an EMR to address workflow and clinical issues and clinician interactions with these systems. It may also be a measure of the current capabilities of EMRs in paediatric settings, rather than a reflection of the organisational commitment to advocating appropriate pain care.

Multiple standard-setting organisations require pain assessment documentation as a key quality indicator (Health Standards Organization, 2023; Schechter et al., 2009). Yet, issues with standardisation and completeness of pain documentation are commonly reported in paediatric hospitals worldwide across various clinical contexts using paper and electronic systems (Andersen et al., 2021; Senger et al., 2021; Vejzovic et al., 2020). Clinicians in this study reported that EMRs provided efficient access to various pain assessment tools that supported consistent pain assessments and contributed to an increase in the frequency of undertaking pain assessment compared to before EMR use. This finding aligns with previous studies in adult hospitals reporting improved frequency of pain assessment documentation among clinicians following the EMR introduction (Gilbertson-White and Shapiro, 2007; Samuels, 2012).

Pain intensity, identified using pain assessment tools, is only one facet of a child’s pain experience and one aspect of the comprehensive pain assessment (Raja et al., 2020). Commensurate with the Lancet Commission’s priority for increasing the visibility of paediatric pain (Eccleston et al., 2021), paediatric pain experts, children, and families have campaigned for EMR and patient portal designs that drive users beyond reporting quantitative pain measures toward capturing broader socio-psychobiological dimensions of pain (Pope et al., 2023). A critical finding in this study was that EMR designs do not currently support clinicians to think, inquire about, or record broader dimensions of acute pain. Relying only on quantitative measures (i.e. pain scores) forsakes understanding pain’s functional, social, and cognitive consequences and contributes to undermanaged pain (Raja et al., 2020) and unsatisfactory hospital experiences for children. To make pain visible, we must harness the potential of EMRs and patient-facing technologies to capture both clinician and patient-generated data on holistic pain experiences. Bringing such pain data to the point of care will offer deeper insights into the complexity of pain, its multifactorial consequences, and the necessity for multimodal interventions.

EMR-based medication interventions can facilitate safe and reproducible prescribing, mitigate variability, and improve prescription quality (Horton et al., 2020; Sutton et al., 2020). For example, a post-tonsillectomy EMR order set that presented four medications (acetaminophen [paracetamol], ibuprofen, oxycodone, and dexamethasone) on postoperative recovery room discharge resulted in standardised and improved pain control regimens and consistency in opioid prescription for children (Horton et al., 2020). Findings from the present study indicate clinicians report that EMRs supported them in the safe administration and management of medications for pain. Still, clinicians reported that EMRs had little influence in steering them toward non-medication interventions. They also reported that they routinely initiate non-medication interventions without documenting them. Our survey questions did not explore why clinicians did not document these interventions. However, the lack of documentation could be partly explained by emerging evidence demonstrating that clinicians are overwhelmed with documentation and alert fatigue with the introduction of EMRs (Van Dort et al., 2021) which have been reported to negatively impact paediatric pain care practices and clinician well-being (Pope et al., 2023).

Our study findings also demonstrate that clinicians spend time double documenting, such as recording pharmacological interventions in free-text progress notes as well as the eMAR. These findings resonate with an adult US hospital EMR audit of 1500 records, revealing duplicate documentation of similar pain care information in two or more places in the EMR in nearly one-third of records (Samuels and Kritter, 2011). Duplicate documentation entries in EMRs between clinician groups across other aspects of inpatient care have also been reported (Laitinen et al., 2014; Törnqvist et al., 2016). Although different sources of pain information may support care continuity, it can also contribute to wasted time and work overload (Gesner et al., 2019). Electronic medical record designs that make it possible to work in integrated ways and with a focus on interdisciplinary pain care are needed to address EMR-associated work overload and information redundancy. Future work could explore the effects of voice recognition and transcription systems on pain care documentation and documentation fatigue.

Research and policy call for the systematic inclusion of children and families as partners in hospitalisations to improve pain-related outcomes (Gatchel et al., 2018; Khadij et al., 2021; Pope et al., 2023; Vasey et al., 2019). Active engagement and shared decision-making throughout the continuum of pain care has been associated with improved communication between the child and family and clinician interdisciplinary teams and improved pain care quality and outcomes (Rao-Gupta et al., 2018; Vasey et al., 2019). Furthermore, when included as partners in care decisions, children and families report feeling more informed, in control and satisfied with pain care, and hopeful about their recovery (Williams et al., 2019). Despite the supporting research and policy, a striking finding in this study was that clinicians perceived EMR designs and interfaces did not sufficiently support family and child active engagement in their pain care. Yet pain clinicians acknowledge the power of having children and families as equal team members fully engaged in person-centred pain care (Ismail et al., 2019; Pope et al., 2023). User-centred approaches that uncover and prioritise addressing barriers and facilitators to child and family inclusion are necessary to guide stakeholder co-design work on novel ways to harness digital technology to facilitate person- and family-centred partnerships in pain care.

### Strengths and limitations

This was the first survey study to examine the reported use of EMRs in paediatric pain care gathered from a sample of experienced health professionals. The recruitment and data collection approach identified diverse participants from several settings who worked with a few of the currently available EMR systems, allowing various perspectives to be represented. Results provided information on clinical practice and recommendations for EMR designs to optimise pain care for hospitalised children and improve outcomes.

Study limitations, which point to directions for future research, are acknowledged. Low response rates and responder bias (i.e. self-selected participants) contribute to bias such that the responses are not considered representative of all paediatric nurses and doctors using EMRs in Australia. Respondents were mostly nurses, with a smaller number of medical doctors, which is a consideration when determining generalisability of the findings. Further research is needed to represent the perspectives of other clinicians involved in the interdisciplinary management of children’s pain. We did not capture information about clinicians’ clinical areas, and the use of EMRs may vary across different clinical areas. For example, responses may vary between emergency departments, medical and surgical inpatient units, and neonatal and paediatric intensive care units. Only five Australian tertiary paediatric hospitals were eligible for inclusion. After this survey, the remaining non-included four of nine Australian tertiary paediatric hospitals have since begun implementing comprehensive EMR systems. The perspectives of clinicians working at these sites remain unknown.

The uneven number of items on the Likert scales may have resulted in midpoint-inflated responses, threatening the validity of inferences made from survey responses. There were some partially completed questionnaires; however, these added to our intelligence for the questions answered. This survey allowed us to easily quantify how clinicians rate particular elements of EMR use in pain care, but we are unable to understand how or why. Therefore, to better understand the individual experience using EMRs in pain care, we advocate for surveys to be complimented with personal narratives and future observational and ethnographic studies combined with EMR data interrogations to validate the clinicians’ reported perceptions. Organisational environmental scanning work is also required to realise clinician-EMR workflow trends, pressures, and issues.

### Implications for practice

Results of this study delineated important recommendations for EMR designs that support clinicians’ understanding of the multidimensionality of children’s pain and the use of multimodal pain treatments. This study shows that efforts should also focus on leveraging EMR and patient portal technologies as collaboration tools to empower children and families and support shared decision-making in pain care.

## Conclusion

This study highlights how hospital EMRs offer tremendous opportunities to improve pain care quality, outcomes, and patient engagement. This survey identified how clinicians currently report using EMRs in caring for hospitalised children with pain. This leads to important recommendations for EMR designs that support clinicians’ understanding of the multidimensionality of children’s pain and that drive comprehensive pain assessment and treatments. Results are directed towards stakeholders who implement and optimise EMRs in acute settings and those who provide pain care to hospitalised paediatric patients. Findings emphasised the importance of future work focused on harnessing patient-facing digital technologies as tools to support children and families as equal team members fully engaged in person-centred pain care. The real test of these systems is not their popularity but whether they produce valuable outcomes for children, families, and clinicians.

## Appendix

### Abbreviation

EMR: Electronic medical records (also known as electronic health records).
